# Supplementary material for: Identification of Oxidative Stress-Related Biomarkers for Pain–Depression Comorbidity Based on Bioinformatics
Source: Int J Mol Sci. 2024 Jul 30;25(15):8353. doi: 10.3390/ijms25158353 (PMC11313298; doi:10.3390/ijms25158353)
Supplement: Supplementary file 1 [file ijms-25-08353-s001.zip › ijms-3123668-supplementary.docx]

**Table S1.** Demographic characteristics.

| **No.** | **Age** | **Gender** | **Ethnicity** | **Diagnosis** | **Pain Scale** | **Groups** |
| --- | --- | --- | --- | --- | --- | --- |
| 1  2  3  4  5  6  7  8  9  10  11  12  13  14  15  16  17  18  19  20  21  22  23  24  25  26  27  28  29  30  31  32  33  34  35  36  37  38  39  40  41  42  43  44  45  46  47  48  49  50  51  52  53  54  55  56  57  58  59  60  61  62  63  64  65  66  67  68  69  70  71  72  73  74  75  76  77  78  79  80  81  82  83  84  85  86  87  88  89  90  91  92  93  94  95  96  97  98  99  100  101  102  103  104  105  106  107  108  109  110  111  112  113  114  115  116  117  118  119  120  121  122  123  124  125  126  127  128  129  130  131  132  133  134  135  136  137  138  139  140  141  142  143  144  145  146  147  148  149  150  151  152  153  154  155  156  157  158  159  160  161  162  163  164  165  166  167  168  169  170  171  172  173  174  175  176  177  178  179  180  181  182  183  184  185  186  187  188  189  190  191  192  193  194  195  196  197  198  199  200  201  202  203  204  205  206  207  208  209  210  211  212  213  214  215  216  217  218  219  220  221  222  223  224  225  226  227  228  229  230  231  232  233  234  235  236  237  238  239  240  241  242  243  244  245  246  247  248  249  250  251  252  253  254  255  256  257  258  259  260  261  262  263  264  265  266  267  268  269  270  271  272  273  274  275  276  277  278  279  280  281  282  283  284  285  286  287  288  289  290  291  292  293  294  295  296  297  298  299  300  301  302  303  304  305  306  307  308  309  310  311  312  313  314  315  316  317  318  319  320  321  322  323  324  325  326  327  328  329  330  331  332  333  334  335  336  337  338  339  340  341  342  343  344  345  346  347  348  349  350  351  352  353  354  355  356  357  358  359  360  361  362  363  364  365  366  367  368  369  370  371  372  373  374  375  376  377  378  379  380  381  382  383  384  385 | 48  52  46  46  48  49  57  54  57  33  38  44  54  26  47  47  29  29  30  51  51  56  44  46  48  42  48  26  26  56  57  62  63  57  58  59  49  51  45  45  45  61  39  59  52  50  49  60  61  37  51  49  54  56  56  52  52  42  52  24  24  43  44  56  58  56  55  54  52  52  53  25  49  61  61  56  55  62  59  62  62  41  38  38  57  57  58  61  56  43  28  61  51  49  50  50  46  46  46  47  51  56  47  47  47  48  64  49  49  50  54  39  40  49  49  42  44  64  54  45  46  67  68  45  28  48  61  62  45  41  48  50  48  65  65  53  45  45  67  43  24  25  43  44  59  44  45  41  48  48  49  50  49  54  54  45  29  38  55  55  43  50  58  55  47  47  52  50  47  51  51  48  48  57  61  51  57  25  26  27  28  54  51  58  58  59  52  37  51  51  52  63  57  58  54  59  56  41  54  54  55  63  52  26  52  55  59  59  45  45  46  54  59  55  50  51  51  51  52  58  58  58  51  54  54  54  52  32  62  36  38  38  34  35  52  40  28  39  55  43  50  39  34  54  54  50  54  57  33  54  36  58  39  54  54  33  56  57  60  54  55  56  46  56  53  46  42  43  42  46  60  60  55  56  45  59  56  56  57  59  49  49  43  24  56  60  60  61  39  39  49  49  50  62  52  52  63  63  59  59  59  61  52  57  57  55  63  42  57  58  62  62  53  51  56  46  48  60  54  56  57  55  56  51  51  53  48  60  61  61  55  61  49  60  51  58  59  61  60  55  60  48  56  59  42  54  55  55  57  47  47  48  56  56  56  54  56  58  54  56  56  51  52  56  63  58  60  48  41  60  59  50  58  38  38  26  47  58  61  62  51  74  50  50  54 | F  M  M  M  M  M  M  M  M  M  F  M  M  F  M  M  M  M  M  M  M  M  M  M  M  F  M  F  F  M  M  M  M  F  F  M  F  M  M  M  M  M  M  M  F  F  F  F  F  M  M  M  M  M  M  M  M  M  M  M  M  F  F  M  M  M  M  M  M  M  M  M  M  M  M  M  M  M  M  M  M  M  M  M  M  M  M  M  M  M  F  M  F  M  M  M  M  M  M  M  M  M  F  F  F  M  M  M  M  M  M  M  M  F  F  M  M  M  M  M  M  M  M  M  F  M  M  M  M  M  M  M  M  M  M  M  M  M  M  M  F  F  M  M  M  M  M  M  M  M  M  M  M  M  M  M  F  M  M  M  M  F  M  M  F  F  F  M  F  F  F  M  M  F  M  M  M  M  M  M  M  M  M  M  M  M  M  M  M  M  M  F  M  M  M  M  F  M  M  M  M  F  M  M  F  M  M  M  M  M  M  M  M  M  M  M  M  M  M  M  M  M  M  M  F  M  F  M  M  M  M  M  M  M  M  F  F  M  M  M  F  M  M  M  M  M  F  F  M  F  F  F  M  M  M  M  F  F  F  M  M  M  M  F  F  M  M  M  M  M  M  M  F  F  F  M  M  M  M  M  M  M  M  M  M  M  M  M  F  F  M  M  M  M  M  M  M  M  M  M  M  M  M  M  M  M  M  M  M  M  M  M  M  M  M  F  F  M  F  M  M  M  M  M  M  M  M  M  M  M  M  M  M  M  M  M  M  F  M  M  F  F  M  M  M  M  M  M  M  M  M  M  M  M  M  F  M  M  F  M  M  M  M  M  M  M  F  F  F  M  M  M  F  F  F  F  M  F  M  M  M  M  M  M  M | Caucasian  African American  Caucasian  Caucasian  African American  African American  Caucasian  Caucasian  African American  African American  Caucasian  Caucasian  African American  Caucasian  Caucasian  Caucasian  Caucasian  Caucasian  Caucasian  Caucasian  Caucasian  Caucasian  Caucasian  African American  African American  Caucasian  African American  Caucasian  Caucasian  Caucasian  Caucasian  Caucasian  Caucasian  Caucasian  Caucasian  African American  Caucasian  African American  Caucasian  Caucasian  Caucasian  African American  Caucasian  African American  Caucasian  Caucasian  Caucasian  African American  African American  Caucasian  African American  Caucasian  Caucasian  African American  African American  Caucasian  Caucasian  Caucasian  Caucasian  African American  African American  Caucasian  Caucasian  African American  African American  African American  Caucasian  African American  Caucasian  Caucasian  Caucasian  African American  Caucasian  Caucasian  Caucasian  African American  African American  Caucasian  Caucasian  Caucasian  Caucasian  Caucasian  Caucasian  Caucasian  Caucasian  Caucasian  Caucasian  Caucasian  Caucasian  Caucasian  Mixed  Caucasian  Caucasian  Caucasian  Caucasian  Caucasian  African American  Caucasian/Native Australian  Caucasian  Caucasian  Caucasian  Caucasian  Caucasian  Caucasian  Caucasian  Caucasian  Caucasian  Caucasian  Caucasian  Caucasian  Caucasian  Hispanic  Hispanic  Caucasian  Caucasian  Caucasian  Caucasian  Caucasian  Caucasian  Caucasian  Caucasian  Caucasian  Caucasian  Caucasian  Caucasian  Caucasian  Caucasian  Caucasian  Caucasian  Caucasian  Caucasian  Caucasian  Caucasian  Caucasian  caucasian  Caucasian  Caucasian  Caucasian  Caucasian  Caucasian  Caucasian  Caucasian  Caucasian  Caucasian  Caucasian  Caucasian  Caucasian  Caucasian  Caucasian  Caucasian  Caucasian  Caucasian  Caucasian  Caucasian  Caucasian  Caucasian  Mixed  Caucasian  Caucasian  Caucasian  Caucasian  Caucasian  Caucasian  Caucasian  Caucasian  Caucasian  Caucasian  Caucasian  Caucasian  Caucasian  Caucasian  Caucasian  Caucasian  Caucasian  Caucasian  Caucasian  African American  Caucasian  Caucasian  Caucasian  Caucasian  Caucasian  Caucasian  African American  African American  Caucasian  Caucasian  Caucasian  Caucasian  Caucasian  Caucasian  African American  African American  African American  Caucasian  African American  Caucasian  Caucasian  Caucasian  Caucasian  Caucasian  African American  Caucasian  Caucasian  Caucasian  Caucasian  African American  African American  Caucasian  Caucasian  Caucasian  African American  African American  Caucasian  African American  African American  African American  African American  African American  Caucasian  African American  Caucasian  African American  African American  Caucasian  African American  Caucasian  Caucasian  Caucasian  African American  Caucasian  Caucasian  Caucasian  Caucasian  Hispanic  Caucasian  Caucasian  Caucasian  African American  Caucasian  Caucasian  Caucasian  Caucasian  African American  African American  African American  Caucasian  Caucasian  Caucasian  Caucasian  African American  Caucasian  Caucasian  Caucasian  Caucasian  Caucasian  Caucasian  Caucasian  African American  Caucasian  Caucasian  Caucasian  African American  Caucasian  Caucasian  African American  African American  African American  African American  African American  Caucasian  Caucasian  African American  African American  Caucasian  African American  African American  African American  African American  African American  Caucasian  Caucasian  Caucasian  Caucasian  African American  African American  African American  African American  Caucasian  Caucasian  African American  African American  African American  Caucasian  African American  African American  Caucasian  Caucasian  African American  African American  African American  African American  African American  African American  Caucasian  African American  Caucasian  Caucasian  Caucasian  Caucasian  Caucasian  Caucasian  African American  Caucasian  Caucasian  Caucasian  Caucasian  Caucasian  African American  Caucasian  Caucasian  Caucasian  African American  Caucasian  Caucasian  Caucasian  African American  African American  African American  African American  African American  African American  African American  African American  African American  African American  African American  Caucasian  African American  African American  Caucasian  African American  Caucasian  Caucasian  Caucasian  African American  African American  African American  African American  African American  African American  African American  Caucasian  African American  African American  African American  Caucasian  Caucasian  African American  Caucasian  Caucasian  African American  African American  African American  African American  African American  Caucasian  African American  Caucasian  Caucasian  Caucasian  African American  Caucasian  Caucasian  Caucasian  Caucasian  Caucasian  Caucasian  Caucasian  Caucasian  African American  Caucasian  African American  African American  African American | MDD  MDD  MDD  MDD  MDD  MDD  MDD  MDD  MDD  MDD  MDD  MDD  MDD  MDD  MDD  MDD  MDD  MDD  MDD  MDD  MDD  MDD  MDD  MDD  MDD  MDD  MDD  MDD  MDD  MDD  MDD  MDD  MDD  MDD  MDD  MDD  MDD  MDD  MDD  MDD  MDD  MDD  MDD  MDD  MDD  MDD  MDD  MDD  MDD  MDD  MDD  MDD  MDD  MDD  MDD  MDD  MDD  MDD  MDD  MDD  MDD  MDD  MDD  MDD  MDD  MDD  MDD  MDD  MDD  MDD  MDD  MDD  MDD  MDD  MDD  MDD  MDD  MDD  BP  BP  BP  BP  BP  BP  BP  BP  BP  BP  BP  BP  BP  BP  BP  BP  BP  BP  BP  BP  BP  BP  BP  BP  BP  BP  BP  BP  BP  BP  BP  BP  BP  BP  BP  BP  BP  BP  BP  BP  BP  BP  BP  BP  BP  BP  BP  BP  BP  BP  BP  BP  BP  BP  BP  BP  BP  BP  BP  BP  BP  BP  BP  BP  BP  BP  BP  BP  BP  BP  BP  BP  BP  BP  BP  BP  BP  BP  BP  BP  BP  BP  BP  BP  BP  BP  BP  BP  BP  BP  BP  BP  BP  BP  BP  BP  BP  BP  BP  BP  BP  BP  BP  BP  BP  BP  BP  BP  BP  BP  BP  BP  BP  BP  BP  BP  BP  BP  BP  BP  BP  BP  BP  BP  BP  BP  BP  BP  MOOD  MOOD  MOOD  MOOD  MOOD  MOOD  MOOD  MOOD  MOOD  MOOD  MOOD  PSYCH  PSYCH  PSYCH  PSYCH  PSYCH  PSYCH  PSYCH  PTSD  PTSD  PTSD  PTSD  PTSD  PTSD  PTSD  PTSD  PTSD  PTSD  PTSD  PTSD  PTSD  PTSD  PTSD  PTSD  PTSD  PTSD  PTSD  PTSD  PTSD  PTSD  PTSD  PTSD  PTSD  PTSD  PTSD  PTSD  PTSD  PTSD  PTSD  PTSD  PTSD  PTSD  PTSD  PTSD  PTSD  PTSD  PTSD  PTSD  PTSD  PTSD  PTSD  PTSD  PTSD  PTSD  SZ  SZ  SZ  SZ  SZ  SZ  SZ  SZ  SZ  SZ  SZ  SZ  SZ  SZ  SZ  SZ  SZ  SZ  SZ  SZ  SZ  SZ  SZ  SZ  SZ  SZ  SZ  SZ  SZ  SZ  SZ  SZ  SZ  SZ  SZ  SZ  SZ  SZ  SZ  SZ  SZ  SZ  SZ  SZ  SZ  SZ  SZ  SZ  SZ  SZ  SZ  SZ  SZ  SZ  SZ  SZ  SZ  SZ  SZ  SZ  SZ  SZ  SZ  SZA  SZA  SZA  SZA  SZA  SZA  SZA  SZA  SZA  SZA  SZA  SZA  SZA  SZA  SZA  SZA  SZA  SZA  SZA  SZA  SZA  SZA  SZA  SZA  SZA  SZA  SZA  SZA  SZA  SZA  SZA  SZA  SZA  SZA  SZA  SZA  SZA  SZA  SZA  SZA  SZA  SZA  SZA  SZA  SZA  SZA  SZA  SZA  SZA  SZA  SZA | 2  2  2  2  0  0  0  0  0  0  0  0  1  1  1  1  1  1  1  1  1  1  1  1  1  2  2  2  2  2  2  2  2  2  2  2  6  6  6.5  7  8  6  7  7  7  7  8  8  8  10  6  6  6  6  6  6  6  6  6  6  6  6  6  7  7  7  7  7  7  7  7  7  8  8  8  8  8  9  0  0  0  0  0  0  0  0  0  1  1  1  2  2  2  0  0  0  0  0  0  0  0  0  0  0  0  0  0  0  0  0  0  0  0  0  0  0  0  0  0  0  0  1  1  1  1  1  1  1  1  1  1  1  1  1  1  1  1  1  2  2  2  2  2  2  2  2  2  2  2  2  2  2  2  2  2  2  6  6  6  6  6  6  7  8  6  6  6  7  7  7  8  8  9  10  6  6  6  6  6  6  6  6  6  6  6  7  7  7  7  7  7  7  7  7  7  7  7  8  8  8  8  8  8  8  8  9  0  0  0  0  1  7  8  10  8  8  9  0  0  0  1  6  8  8  0  0  1  2  0  0  0  0  0  0  0  0  1  1  2  2  2  2  2  6  6  6.5  7  7  7  10  7  7.5  6  6  6  6  6  6  6  7  7  7  7  7  8  8  8  8  8  9  0  0  0  0  2  0  0  0  0  0  0  0  0  0  0  0  0  0  0  0  0  0  0  0  0  0  0  0  0  0  0  0  0  1  1  1  1  2  2  2  2  2  2  2  2  6  6  7  7  7  7  8  6  6  6  6  6  6  6  7  8  8  9  0  0  0  0  1  2  2  2  2  0  0  0  0  0  0  0  0  0  0  1  1  1  1  1  1  2  2  2  7  7  7  8  8  8  8  6  9  10  6  6  6  6  7  7  7  7  7  7  8  8  10  2 | Low Pain  Low Pain  Low Pain  Low Pain  Low Pain  Low Pain  Low Pain  Low Pain  Low Pain  Low Pain  Low Pain  Low Pain  Low Pain  Low Pain  Low Pain  Low Pain  Low Pain  Low Pain  Low Pain  Low Pain  Low Pain  Low Pain  Low Pain  Low Pain  Low Pain  Low Pain  Low Pain  Low Pain  Low Pain  Low Pain  Low Pain  Low Pain  Low Pain  Low Pain  Low Pain  Low Pain  High Pain  High Pain  High Pain  High Pain  High Pain  High Pain  High Pain  High Pain  High Pain  High Pain  High Pain  High Pain  High Pain  High Pain  High Pain  High Pain  High Pain  High Pain  High Pain  High Pain  High Pain  High Pain  High Pain  High Pain  High Pain  High Pain  High Pain  High Pain  High Pain  High Pain  High Pain  High Pain  High Pain  High Pain  High Pain  High Pain  High Pain  High Pain  High Pain  High Pain  High Pain  High Pain  Low Pain  Low Pain  Low Pain  Low Pain  Low Pain  Low Pain  Low Pain  Low Pain  Low Pain  Low Pain  Low Pain  Low Pain  Low Pain  Low Pain  Low Pain  Low Pain  Low Pain  Low Pain  Low Pain  Low Pain  Low Pain  Low Pain  Low Pain  Low Pain  Low Pain  Low Pain  Low Pain  Low Pain  Low Pain  Low Pain  Low Pain  Low Pain  Low Pain  Low Pain  Low Pain  Low Pain  Low Pain  Low Pain  Low Pain  Low Pain  Low Pain  Low Pain  Low Pain  Low Pain  Low Pain  Low Pain  Low Pain  Low Pain  Low Pain  Low Pain  Low Pain  Low Pain  Low Pain  Low Pain  Low Pain  Low Pain  Low Pain  Low Pain  Low Pain  Low Pain  Low Pain  Low Pain  Low Pain  Low Pain  Low Pain  Low Pain  Low Pain  Low Pain  Low Pain  Low Pain  Low Pain  Low Pain  Low Pain  Low Pain  Low Pain  Low Pain  Low Pain  Low Pain  High Pain  High Pain  High Pain  High Pain  High Pain  High Pain  High Pain  High Pain  High Pain  High Pain  High Pain  High Pain  High Pain  High Pain  High Pain  High Pain  High Pain  High Pain  High Pain  High Pain  High Pain  High Pain  High Pain  High Pain  High Pain  High Pain  High Pain  High Pain  High Pain  High Pain  High Pain  High Pain  High Pain  High Pain  High Pain  High Pain  High Pain  High Pain  High Pain  High Pain  High Pain  High Pain  High Pain  High Pain  High Pain  High Pain  High Pain  High Pain  High Pain  High Pain  Low Pain  Low Pain  Low Pain  Low Pain  Low Pain  High Pain  High Pain  High Pain  High Pain  High Pain  High Pain  Low Pain  Low Pain  Low Pain  Low Pain  High Pain  High Pain  High Pain  Low Pain  Low Pain  Low Pain  Low Pain  Low Pain  Low Pain  Low Pain  Low Pain  Low Pain  Low Pain  Low Pain  Low Pain  Low Pain  Low Pain  Low Pain  Low Pain  Low Pain  Low Pain  Low Pain  High Pain  High Pain  High Pain  High Pain  High Pain  High Pain  High Pain  High Pain  High Pain  High Pain  High Pain  High Pain  High Pain  High Pain  High Pain  High Pain  High Pain  High Pain  High Pain  High Pain  High Pain  High Pain  High Pain  High Pain  High Pain  High Pain  High Pain  Low Pain  Low Pain  Low Pain  Low Pain  Low Pain  Low Pain  Low Pain  Low Pain  Low Pain  Low Pain  Low Pain  Low Pain  Low Pain  Low Pain  Low Pain  Low Pain  Low Pain  Low Pain  Low Pain  Low Pain  Low Pain  Low Pain  Low Pain  Low Pain  Low Pain  Low Pain  Low Pain  Low Pain  Low Pain  Low Pain  Low Pain  Low Pain  Low Pain  Low Pain  Low Pain  Low Pain  Low Pain  Low Pain  Low Pain  Low Pain  Low Pain  Low Pain  Low Pain  Low Pain  Low Pain  High Pain  High Pain  High Pain  High Pain  High Pain  High Pain  High Pain  High Pain  High Pain  High Pain  High Pain  High Pain  High Pain  High Pain  High Pain  High Pain  High Pain  High Pain  Low Pain  Low Pain  Low Pain  Low Pain  Low Pain  Low Pain  Low Pain  Low Pain  Low Pain  Low Pain  Low Pain  Low Pain  Low Pain  Low Pain  Low Pain  Low Pain  Low Pain  Low Pain  Low Pain  Low Pain  Low Pain  Low Pain  Low Pain  Low Pain  Low Pain  Low Pain  Low Pain  Low Pain  High Pain  High Pain  High Pain  High Pain  High Pain  High Pain  High Pain  High Pain  High Pain  High Pain  High Pain  High Pain  High Pain  High Pain  High Pain  High Pain  High Pain  High Pain  High Pain  High Pain  High Pain  High Pain  High Pain  Low Pain |

M males, F females, MDD major depressive disorder, BP bipolar, MOOD mood disorder, PSYCH schizophrenia and schizoaffective combined, PTSD posttraumatic stress disorder, SZ schizophrenia, SZA schizoaffective.

**Table S2.** Identified key genes.

| **Detail** | **WGCNA-yellow** | **DEOSRGs** | **DEGs** | **SharedSets** |
| --- | --- | --- | --- | --- |
| DNAJC3  FOS  ABHD5  ADGRE3  ADGRE5  AGO4  ARAP3  ARHGAP25  ARHGEF40  BAZ2B-AS1  BCL6  DHRS13  EGLN1  ETS2  IL13RA1  IRAG1  KIAA0232  KRT23  LINC01127  MGAM  MMP25  MYADM  NHSL2  NRBF2  ORM1  PPP1R12B  PPP4R1  PREX1  PYGL  RAPGEF2  REPS2  RNF24  SH3RF3  SIGLEC5  SKAP2  SLC45A4  SLC6A6  ST6GALNAC2  STX3  SVIL  TECPR2  TKT  TSEN34 | 1  1  1  1  1  1  1  1  1  1  1  1  1  1  1  1  1  1  1  1  1  1  1  1  1  1  1  1  1  1  1  1  1  1  1  1  1  1  1  1  1  1  1 | 1  1  0  0  0  0  0  0  0  0  0  0  0  0  0  0  0  0  0  0  0  0  0  0  0  0  0  0  0  0  0  0  0  0  0  0  0  0  0  0  0  0  0 | 1  1  1  1  1  1  1  1  1  1  1  1  1  1  1  1  1  1  1  1  1  1  1  1  1  1  1  1  1  1  1  1  1  1  1  1  1  1  1  1  1  1  1 | 3  3  2  2  2  2  2  2  2  2  2  2  2  2  2  2  2  2  2  2  2  2  2  2  2  2  2  2  2  2  2  2  2  2  2  2  2  2  2  2  2  2  2 |
